# Unsupervised Clustering Analysis Based on MODS Severity Identifies Four Distinct Organ Dysfunction Patterns in Severely Injured Blunt Trauma Patients

**DOI:** 10.3389/fmed.2020.00046

**Published:** 2020-02-25

**Authors:** Dongmei Liu, Rami A. Namas, Yoram Vodovotz, Andrew B. Peitzman, Richard L. Simmons, Hong Yuan, Qi Mi, Timothy R. Billiar

**Affiliations:** ^1^Department of Cardiology, Third Xiangya Hospital of Central South University, Changsha, China; ^2^Department of Surgery, University of Pittsburgh, Pittsburgh, PA, United States; ^3^Center for Inflammation and Regenerative Modeling, McGowan Institute for Regenerative Medicine, University of Pittsburgh, Pittsburgh, PA, United States; ^4^Department of Sports Medicine and Nutrition, University of Pittsburgh, Pittsburgh, PA, United States

**Keywords:** multiple organ dysfunction, intensive care unit, blunt trauma, biomarkers, fuzzy C-means clustering, dynamic network analysis

## Abstract

**Purpose:** We sought to identify a MODS score parameter that highly correlates with adverse outcomes and then use this parameter to test the hypothesis that multiple severity-based MODS clusters could be identified after blunt trauma.

**Methods:** MOD score across days (D) 2–5 was subjected to Fuzzy C-means Clustering Analysis (FCM) followed by eight Clustering Validity Indices (CVI) to derive organ dysfunction patterns among 376 blunt trauma patients admitted to the intensive care unit (ICU) who survived to discharge. Thirty-one inflammation biomarkers were assayed (Luminex™) in serial blood samples (3 samples within the first 24 h and then daily up to D 5) and were analyzed using Two-Way ANOVA and Dynamic Network analysis (DyNA).

**Results:** The FCM followed by CVI suggested four distinct clusters based on MOD score magnitude between D2 and D5. Distinct patterns of organ dysfunction emerged in each of the four clusters and exhibited statistically significant differences with regards to in-hospital outcomes. Interleukin (IL)-6, MCP-1, IL-10, IL-8, IP-10, sST2, and MIG were elevated differentially over time across the four clusters. DyNA identified remarkable differences in inflammatory network interconnectivity.

**Conclusion:** These results suggest the existence of four distinct organ failure patterns based on MOD score magnitude in blunt trauma patients admitted to the ICU who survive to discharge.

## Introduction

Trauma remains the leading cause of mortality and morbidity for individuals under 55 years and accounts for 30% of all life-years lost, with over 190,000 lives lost annually in the USA (1, 2). With advances in prehospital transport and resuscitation strategies, patterns of traumatic death have significantly changed over the past three decades (3–5). Trauma-related deaths have now assumed a largely bimodal distribution, with a vast majority of deaths occurring at the scene or within the first day after injury as a consequence of massive head injury or uncontrolled bleeding (5, 6). However, patients who survive beyond the initial traumatic insult are prone to develop a state of persistent critical illness manifested by prolonged intensive care unit (ICU) and hospital length of stays (LOS), and a persistent risk of late in-hospital and post-discharge complications (7–10).

A common central factor contributing to outcomes following injury is the accompanying immuno-inflammatory response. If this response is appropriate in magnitude and duration it can aid in re-establishing host homeostasis. However, a dysregulated response is associated with multiple organ dysfunction syndrome (MODS) which can evolve to a state of persistent critical illness and a continued increased risk for complications and death after discharge (11–13). Trauma-induced MODS is widely believed to be the leading cause of death among ICU patients being responsible for 50–80% of ICU mortality (14, 15). Typically, MODS peaks within 5 days of injury and is associated with a complicated clinical course; however, the type and number of distinct organ failure patterns that occur after injury are not known. Hence, the current challenge in the early management of severely injured blunt trauma patients is to predict and then prevent MODS (16–18). However, to do this, there is a need to define the patterns of organ dysfunction in trauma patients that survive the early mortality window and whether distinct MODS patterns are associated with identifiable differences in the early systemic inflammatory response.

In the current study, we utilized an unsupervised clustering strategy to identify the number of MODS-based phenotypes that followed severe blunt injury in a cohort of 493 trauma patients admitted to the ICU. To optimize our study cohort, patients discharged prior to day 5 from the ICU and 21 patients who died before discharge were excluded, which yielded a total of 376 patients used in the current study. The MOD score magnitude over days 2–5 was found to correlate well with adverse in-hospital outcomes and was used to identify four distinct severity-based MODS clusters. The four clusters exhibited differential early inflammation biomarker profiles and correlated with subsequent in-hospital adverse outcomes. These findings provide evidence for the emergence of multiple definable organ dysfunction patterns after severe blunt injury. This information can be useful for the identification of prognostic variables to predict organ dysfunction severity and patterns following blunt traumatic injury.

## Materials and Methods

### Patient Enrollment, Sampling, and Data Collection

Blunt trauma patients deemed eligible for enrollment were at least 18 years of age at time of the trauma, admitted to the ICU as part of the post-trauma management, and were expected to survive beyond the initial 24 h post-injury as per the on-call trauma surgeon. Reasons for ineligibility were isolated head injury or brain death criteria, or pregnancy. Three plasma samples were collected within the first 24 h following injury, as follows: (1) the initial blood draw upon arrival within 4 h from time of injury; (2) within 4–12 h of admission to the emergency department (ED); and (3) at 24 h of ED admission. Subsequent samples were obtained from day (D) 1 to D5 post-injury. Demographic and clinical data were collected from the inpatient electronic medical record and the trauma registry database. The clinical database and biobank were maintained prospectively from 2004 to 2012, for a total of 8 years period. Totally 493 patients were enrolled in the observational study. The Marshall Multiple Organ Dysfunction (MOD) score (19) was calculated daily during the patients' ICU stay.

### Optimization of Study Cohort

Organ failure is known to peak within the first 5 days after injury (20). Therefore, out of the 493 patients enrolled in the observational study (who were admitted to the ICU of the UPMC Presbyterian University Hospital, a Level 1 trauma center), we identified a subset of patients (*n* = 376) with complete sequential MOD score data who remained in the ICU for at least 5 days post-injury. Patients were excluded because they were discharged from the ICU prior to 5 days and therefore had incomplete MOD score data (*n* = 96) or because they died prior to hospital discharge (*n* = 21) and therefore the incidence of in hospital complication rates could not be assessed. The patients that died in-hospital have been previously described in detail (21) and are referenced in this study as a separate group.

### Fuzzy C-Means Clustering

To identify the number of distinct MOD score severity-based clusters present in the first 5 days after injury, the sequential MOD scores across days 2–5 for the 376 patients admitted to the ICU after trauma were subjected to fuzzy C-means (FCM) clustering. The FCM is a soft partition, unsupervised clustering method that allows each piece of data to belong to more than one cluster (22). The FCM assigns membership values to each of the data points that indicate the degree to which the data points belong to the different clusters. This feature, in some degree, fits the characteristic of heterogeneous clinical data that exhibits no clear boundaries between clusters. The objective function of the FCM algorithm is to minimize the objective function (see below):

$$\begin{array}{l} J\left(U,V\right)=\overset{n}{\underset{i=1}{\sum }}\overset{c}{\underset{j=1}{\sum }}{\left(\mu_{ij}\right)}^{m}{∥x_{i}-v_{j}∥}^{2} \end{array}$$

Where X = {*x*_1_, …, *x*_*n*_} is a collection of n elements. In our case, n = 376, and *x*_*i*_ represents a vector of consisting of *i*th patient's four MOD scores from days 2–5. μ_*ij*_ ∈ [0, 1] is the membership of *x*_*i*_ to *j*th cluster, m represents the fuzzifier parameter (set up as 2), *c* is the number of clusters with cluster centers V = {*v*_1_, …, *v*_*c*_}, and ∥ *x*_*i*_ − *v*_*j*_ ∥ represents the distance of *x*_*i*_ to the center of *j*th cluster.

As described in the objective function, the number of clusters (*c*) needs to be preset. To define the optimal number of clusters, we performed FCM based on the Euclidean distance with *c* set from 2 (lowest) to 6 (highest) clusters. Next, to determine the optimal number of clusters, we utilized eight internal Clustering Validity Indices (CVI), which evaluate the goodness of a clustering structure relying only on the information in the data and allow for the quantification of intra-cluster compactness or inter-cluster separation (23, 24). The CVI analysis included: C index, Dunn index, Gamma index, GDI index, G_plus index, PBM index, S_DBW index, and Tau index (further details of the clustering validity indices are available at: https://cran.r-project.org/web/packages/clusterCrit/vignettes/clusterCrit.pdf). For each index, specific rule of either computing the greatest index value (max) or the smallest index value (min) must be applied in order to determine the best index value for optimal partition. Among the CVIs aforementioned, the best partition is the one corresponding to the “min” value of C index, G-plus index, S-DBW index and the “max” value of indices including Dunn index, Gamma index, GDI index, PBM index, and Tau index. These analyses were carried out using R (The R Project for Statistical Computing, Version 3.2.2).

### Analysis of Inflammation Biomarkers

Blood samples were collected into citrated tubes via central venous or arterial catheters within 24 h of admission and daily up to 5 days post-injury. The blood samples were centrifuged, and plasma was aliquoted and stored at −80°C for subsequent analysis of inflammatory mediators (a total of 30 biomarkers). The human inflammatory MILLIPLEX™ MAP Human Cytokine/Chemokine Panel-Premixed 26 Plex, MILLIPLEX™ MAP Human Th17 Panel (Millipore Corporation, Billerica, MA), Luminex™ 100 IS analyzer (Luminex, Austin, TX), and MAGPIX^®^ system (MilliporeSigma, Austin, TX) were used to measure plasma levels of interleukin (IL)-1β, IL-1 receptor antagonist (IL-1RA), IL-2, soluble IL-2 receptor-α (sIL-2Rα), IL-4, IL-5, IL-6, IL-7, IL-8 (CCL8), IL-9, IL-10, IL-13, IL-15, IL-17A, IL-17E/IL-25, IL-21, IL-22, IL-23, IL-33, interferon (IFN)-γ, IFN-α, IFN-γ inducible protein (IP)-10 (CXCL10), monokine induced by gamma interferon (MIG; CXCL9), macrophage inflammatory protein (MIP)-1α (CCL3), MIP-1β (CCL4), monocyte chemotactic protein (MCP)-1 (CCL2), granulocyte-macrophage colony stimulating factor (GM-CSF), Eotaxin (CCL11), and tumor necrosis factor alpha (TNF-α). The Luminex™ system was used in accordance to manufacturer's instructions. Plasma levels of soluble ST2 (sST2) were measured by ELISA according to the manufacturers' instructions (R&D Systems, Minneapolis, MN).

### Statistical Analysis

All data are expressed as mean ± SEM. Statistical analysis between groups was performed by One-way Analysis of Variance (ANOVA) followed by Tukey *post-hoc* analysis using SigmaPlot™ 11 software (Systat Software, Inc., San Jose, CA). Fisher's exact test was performed for categorical data using Graphpad PRISM (GraphPad Software, Inc., La Jolla, CA). Group-time interaction of plasma inflammatory mediators' levels was determined by Two-Way ANOVA. *P* < 0.05 was considered statistically significantly different for all analyses. Dynamic Network Analysis (DyNA) was performed to gain insights into the temporal dynamic changes in network connectivity of the post-traumatic inflammatory response [as we have shown previously (8, 21, 25)] among the FCM-defined clusters.

## Results

### Clustering Validity Indices Identify Four Distinct MOD Score Clusters Following Severe Blunt Trauma

**Figure 1:**
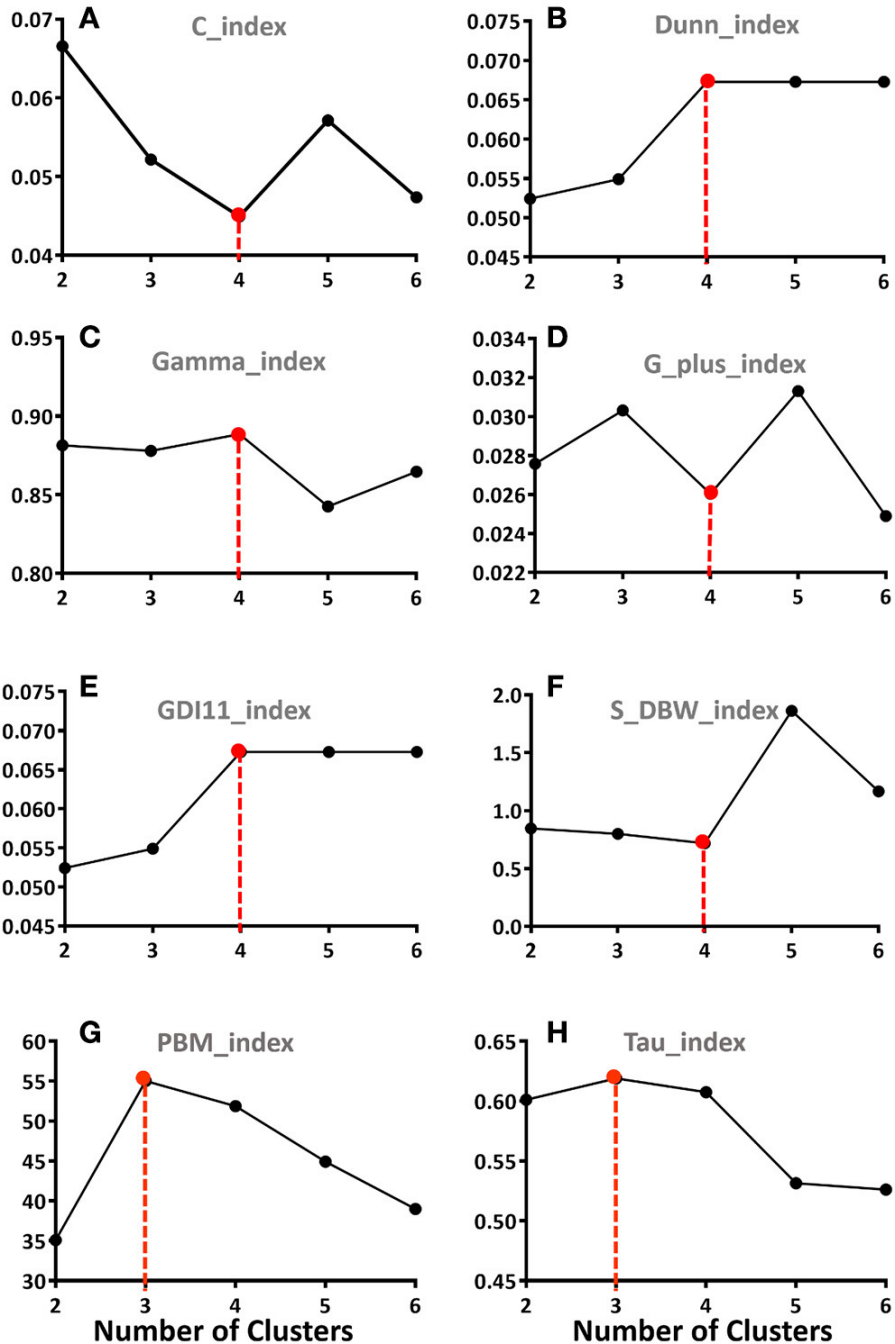
Eight separate clustering validation indices (CVI) were used to define the number of potential distinct MODS Clusters using the average MOD score data from day 2 through day 5 post-injury. The CVI analysis included: **(A)** C index; **(B)** S-DBW index; **(C)** Dunn index; **(D)** Gamma index, **(E)** G-plus index; **(F)** GDI index; **(G)** PBM index; and **(H)** Tau index. Six indices **(A–F)** indicated that four Clusters was the optimal number, while two indices **(G,H)** suggested three Clusters.

**Figure 2:**
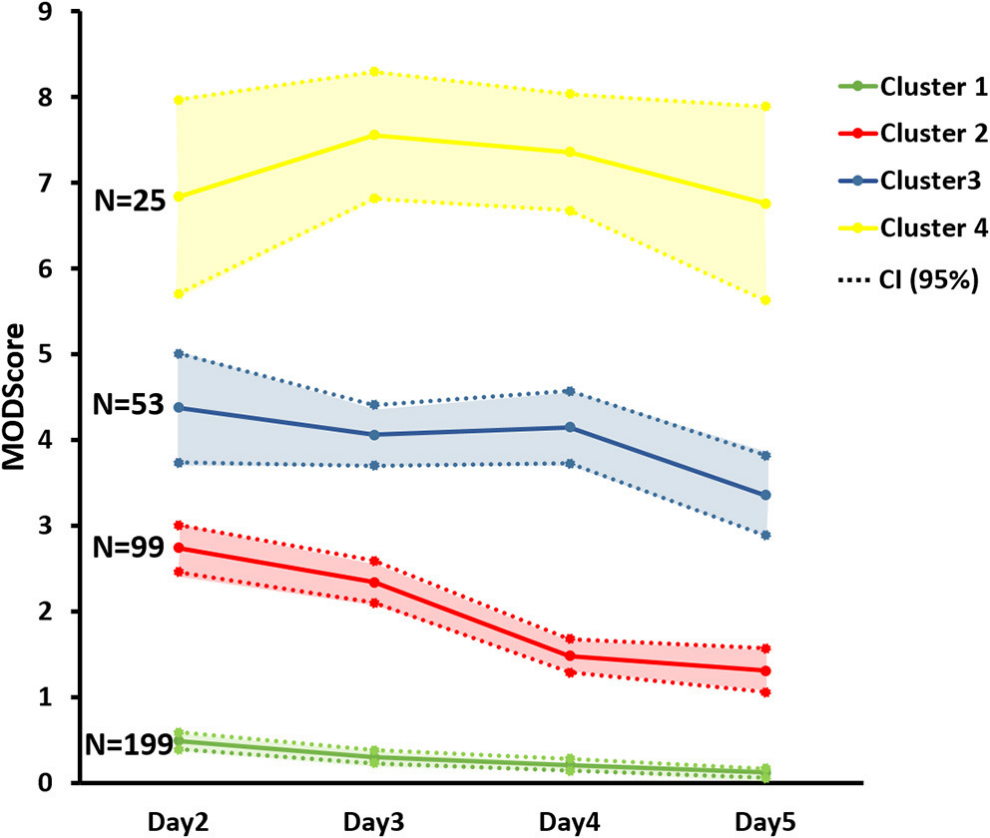
Average MOD scores of the four FCM-derived Clusters from day 2 through day 5 post-injury based on the fuzzy C-means clustering analysis. Data presented as mean with 95% confidence interval (CI).

We focused on days 2–5 because this incorporates the known peak in MODS post-injury (20) but avoids the impact of inadequate resuscitation on MOD score sometimes observed in the first 24 h. To determine the number of distinct severity-based MODS clusters that appear after blunt injury, the MOD score data across D2–D5 were subjected to Fuzzy Clustering Analysis followed by eight separate clustering validity indices. Six indices indicated that four clusters was the optimal number, while two indices suggested three clusters (Figure 1). Based on these analyses, FCM segregated the patient cohort into the following four clusters: Cluster 1 (*n* = 199, mean MOD score = 0.28 ± 0.02), Cluster 2 (*n* = 99, mean MOD score = 1.97 ± 0.07), Cluster 3 (*n* = 53, mean MOD score = 3.99 ± 0.12), and Cluster 4 (*n* = 25, mean MOD score = 7.13 ± 0.23). The MOD score values on individual days were statistically different between the four clusters as determined by Two-way ANOVA (Figure 2).

### The Four MOD Score Clusters Differed in Injury Patterns and Presentation Characteristics

In terms of overall demographics, there was no statistically significant difference in average age or gender distribution among the four clusters (Table 1). However, Cluster 1 had a statistically significantly lower average injury severity score (ISS) than Clusters 2–4 (*P*_2vs.1_ = 0.041; *P*_3vs.1_ < 0.001). There was no statistical difference in ISS between Clusters 2, 3, and 4. To determine if injury patterns differed between the clusters, the abbreviated injury scale for six body regions were compared (Table 1). Cluster 2 exhibited greater rates of abdominal and extremity injuries (*P* = 0.014 and *P* = 0.003, respectively) when compared to Cluster 1. Patients in Cluster 3 had higher head and neck injury scores when compared to Cluster 1 and 2 (*P* = 0.011 and 0.02, respectively) and a statistically significantly higher incidence of brain injury than Cluster 1 (*P* = 0.003) and Cluster 2 (*P* = 0.010). There was no difference in brain injury rates between Clusters 3 and 4 (Table 1). Thus, Cluster 1 patients were less severely injured while Clusters 3 and 4 included patients that were more likely to have traumatic brain injury.

**Table 1:** Demographics and injury pattern characteristics among the four Clusters (Cluster 1, *n* = 199; Cluster 2, *n* = 99; Cluster 3, *n* = 53; and Cluster 4, *n* = 25).

	**Cluster 1 (*n* = 199)**	**Cluster 2 (*n* = 99)**	**Cluster 3 (*n* = 53)**	**Cluster 4 (*n* = 25)**	***P*****-value^s^**		
					***P* (1 vs. 2)**	***P* (2 vs. 3)**	***P* (3 vs. 4)**
**DEMOGRAPHICS**							
Age, year^a^	49.4 ± 1.3	47.6 ± 1.8	47.5 ± 3.0	46.3 ± 4.2	0.76		
Gender, M/F	134/65	70/29	36/17	20/5	0.61		
Injury severity score (ISS)^a^	18.7 ± 0.7	22.1 ± 1.1	26.0 ± 1.7	24.3 ± 2.6	0.04^s^	0.12	0.90
**ABBREVIATED INJURY SCALE (AIS)**							
S1 (head/neck)^a^	1.19 ± 0.12	1.15 ± 0.18	2.09 ± 0.28	1.84 ± 0.09	1	0.02^s^	0.95
S2 (face)^a^	0.42 ± 0.06	0.43 ± 0.09	0.36 ± 0.11	0.32 ± 0.14	0.9		
S3 (chest)^a^	2.17 ± 0.11	2.25 ± 0.16	2.43 ± 0.26	2.16 ± 0.34	0.73		
S4 (abdomen)^a^	1.02 ± 0.10	1.56 ± 0.15	1.64 ± 0.22	1.28 ± 0.32	0.01^s^	0.99	0.73
S5 (extremities)^a^	1.36 ± 0.09	1.90 ± 0.13	2.26 ± 0.13	1.72 ± 0.27	0.003^s^	0.31	0.27
S6 (external)^a^	0.69 ± 0.04	0.67 ± 0.06	0.70 ± 0.08	0.60 ± 0.12	0.88		
**BRAIN INJURY**							
Brain injury, *n* (%)	34 (17.1%)	17 (17.2%)	19 (35.8%)	8 (32.0%)	0.99	0.01 s	0.74

a *Values are expressed as mean ± SEM*.

One-Way ANOVA or Fisher exact test were used as appropriate with statistical significance set at

s *P < 0.05*.

Next, we identified the differences in physiologic and biochemical data on presentation among the four MOD score clusters. Clusters 2–4 had lower average systolic blood pressures and hemoglobin levels upon presentation when compared to patients in Cluster 1 (Table 2). Patients in Cluster 3 had higher blood creatinine levels on admission compared to Clusters 1 and 2. Admission coagulation parameters (Prothrombin time, International Normalized Ratio, and Partial Thromboplastin Time) were higher in Cluster 3 (16.0 ± 0.7, *P* = 0.007; 1.36 ± 0.07, *P* = 0.001; 29.0 ± 1.2, *P* = 0.003; respectively) and Cluster 4 (16.3 ± 0.8, *P* = 0.03; 1.35 ± 0.09, *P* = 0.037; 28.3 ± 0.7, *P* = 0.23; respectively) compared to Cluster 1 (14.4 ± 0.2; 1.16 ± 0.02; 26.3 ± 0.3; respectively) (Table 2). Therefore, patients in Clusters 2–4 were more likely to be in shock at admission while patients in Clusters 3 and 4 were more likely to present with evidence of renal dysfunction and coagulation abnormalities.

**Table 2:** Physiological and biochemical parameters among the four Clusters (Cluster 1, *n* = 199; Cluster 2, *n* = 99; Cluster 3, *n* = 53; and Cluster 4, *n* = 25).

	**Cluster 1 (*n* = 199)**	**Cluster 2 (*n* = 99)**	**Cluster 3 (*n* = 53)**	**Cluster 4 (*n* = 25)**	***P*****-value^s^**		
					***P* (1 vs. 2)**	***P* (2 vs. 3)**	***P* (3 vs. 4)**
Heart rate	93.1 ± 1.5	98.2 ± 2.5	96.7 ± 3.2	99.9 ± 5.6	0.25		
Systolic blood pressure	130.1 ± 1.9	115.0 ± 3.1	116.2 ± 4.6	110.4 ± 6.0	<0.001^s^	0.99	0.85
Shock index	0.8 ± 0.1	1.0 ± 0.1	1.0 ± 0.1	1.0 ± 0.1	0.07		
Base deficit (BD)	4.6 ± 0.4	5.7 ± 0.4	6.5 ± 0.6	6.4 ± 0.7	0.27	0.65	0.99
Lactate	2.7 ± 0.2	2.8 ± 0.2	3.3 ± 0.3	3.6 ± 0.3	0.87	0.41	0.88
Creatinine phosphokinase (CPK)	1047 ± 177	2910 ± 944	1873 ± 393	4673 ± 1883	0.19	0.80	0.22
Sodium (Na)	138.2 ± 0.2	138.0 ± 0.4	139.3 ± 0.4	138.1 ± 0.7	0.09		
Potassium (K)	4.0 ± 0.0	3.8 ± 0.1	4.0 ± 0.1	3.9 ± 0.2	0.15		
Chloride (Cl)	105.6 ± 0.3	107.1 ± 0.6	107.6 ± 0.7	107.9 ± 0.9	0.10	0.94	0.99
Partial pressure of arterial carbon dioxide (PaCO_2_)	23.4 ± 0.2	22.4 ± 0.4	22.0 ± 0.6	22.0 ± 0.2	0.06	0.93	1.00
Anion gap	10.2 ± 0.3	9.5 ± 0.4	10.2 ± 0.6	9.0 ± 0.7	0.31		
Blood urea nitrogen (BUN)	14.5 ± 0.4	14.7 ± 0.7	16.9 ± 1.7	14.0 ± 0.9	0.17		
Creatinine	1.01 ± 0.05	1.05 ± 0.04	1.37 ± 0.13	1.24 ± 0.06	0.92	0.01^s^	0.82
Glucose	152.7 ± 4.2	158.0 ± 6.6	154.8 ± 7.4	189.2 ± 12.9	0.89	0.99	0.09
Albumin	3.5 ± 0.1	3.1 ± 0.1	3.2 ± 0.2	3.1 ± 0.3	0.06		
Total protein	5.9 ± 0.2	5.6 ± 0.2	5.4 ± 0.3	4.7 ± 0.5	0.57	0.93	0.62
Bilirubin total	0.8 ± 0.1	1.3 ± 0.2	0.9 ± 0.1	1.4 ± 0.4	0.05	0.28	0.58
Bilirubin direct	1.4 ± 1.2	0.3 ± 0.0	0.3 ± 0.1	0.6 ± 0.2	0.80		
Alanine amino-transferase (ALT)	111.4 ± 21.0	125.4 ± 32.9	121.0 ± 40.2	140.6 ± 47.6	0.97		
Aspartate amino-transferase (AST)	148.5 ± 29.4	157.2 ± 32.9	187.5 ± 41.6	185.3 ± 57.9	0.88		
White blood cell count	15.4 ± 0.4	15.9 ± 0.7	16.5 ± 1.2	16.2 ± 1.4	0.67		
Hemoglobin	13.3 ± 0.1	12.5 ± 0.2	12.3 ± 0.3	12.8 ± 0.4	0.01^s^	0.98	0.82
Hematocrit	38.7 ± 0.4	36.5 ± 0.5	36.1 ± 0.9	37.1 ± 1.2	0.01^s^	0.97	0.87
Platelets	244.8 ± 5.1	221.7 ± 8.4	222.3 ± 10.5	199.8 ± 14.7	0.07	1.00	0.61
Neutrophils	75.7 ± 0.8	74.4 ± 1.2	73.0 ± 1.8	73.5 ± 2.4	0.41		
Lymphs	14.9 ± 0.7	16.1 ± 1.1	17.6 ± 1.7	16.0 ± 1.6	0.36		
Monocytes	5.8 ± 0.2	5.8 ± 0.2	5.4 ± 0.4	4.6 ± 0.4	0.06		
Eosinophils	0.96 ± 0.08	0.83 ± 0.11	0.97 ± 0.18	0.67 ± 0.21	0.56		
Basophils	0.17 ± 0.03	0.18 ± 0.04	0.28 ± 0.07	0.08 ± 0.06	0.24		
Prothrombin time (PT), seconds	14.4 ± 0.2	15.0 ± 0.3	16.0 ± 0.7	16.3 ± 0.8	0.38		
International normalized ratio (INR)	1.16 ± 0.02	1.25 ± 0.03	1.36 ± 0.07	1.35 ± 0.09	0.13	0.27	0.99
Partial thromboplastin time (PTT), seconds	26.3 ± 0.3	27.3 ± 0.5	29.0 ± 1.2	28.3 ± 0.7	0.37	0.20	0.99

*Values are expressed as mean ± SEM*.

One-Way ANOVA used with statistical significance set at

s *P < 0.05*.

### MOD Score Clusters Differ in Clinical Outcomes

There were statistically significant differences among the four clusters with regards to in-hospital outcomes, including ICU and total hospital length of stay (LOS), days on mechanical ventilation as well as the incidence of NI being all greatest in Clusters 3 and 4 (Table 3). Surgical intervention rates (within the first 24 h-all types of procedures) were lowest in Cluster 1 and were significantly different between Cluster 1 and Clusters 2–4 (Table 3). Patients in Cluster 4 were more likely to require a vascular intervention. Patients in Cluster 2–4 were more likely to receive a transfusion in the first 24 h than patients in Cluster 1 and patients in Cluster 4 received significantly greater volumes of packed red blood cells (PRBC) and fresh frozen plasma (FFP) when compared to patients in Clusters 1–3 (Table 3). These findings further establish that patients that fall into Cluster 1 have less severe injuries than patients in the other clusters, while patients in Cluster 4 are distinguished by a greater need for both PRBC and FFP.

**Table 3:** Clinical course among the four Clusters (Cluster 1, *n* = 199; Cluster 2, *n* = 99; Cluster 3, *n* = 53; and Cluster 4, *n* = 25).

	**Cluster 1 (*n* = 199)**	**Cluster 2 (*n* = 99)**	**Cluster 3 (*n* = 53)**	**Cluster 4 (*n* = 25)**	***P*****-value^s^**		
					**P (1 vs. 2)**	**P (2 vs. 3)**	**P (3 vs. 4)**
**SURGICAL INTERVENTIONS WITHIN 24 H**							
Interventions within 24 h, *n* (%)	64 (32%)	57 (59%)	34 (64%)	16 (64%)	<0.001^s^	0.57	0.99
Laparotomy, *n* (%)	16 (8%)	27 (28%)	18 (34%)	10 (40%)	<0.001^s^	0.43	0.60
Orthopedic, *n* (%)	50 (25%)	46 (47%)	26 (49%)	11 (44%)	<0.001^s^	0.85	0.68
Vascular, *n* (%)	7 (4%)	12 (12%)	4 (8%)	8 (32%)	0.004^s^	0.36	0.02^s^
Craniotomy, *n* (%)	8 (4%)	4 (4%)	4 (8%)	0 (0%)	0.51		
Thoracic, *n* (%)	2 (1%)	5 (5%)	3 (6%)	4 (16%)	0.04^s^	1	0.20
**BLOOD TRANSFUSION WITHIN 24 H**							
Blood transfusion, *n* (%)	33 (17%)	34 (34%)	25 (47%)	13 (52%)	0.001^s^	0.12	0.69
PRBC	782 ± 157	1906 ± 352	2061 ± 615	3381 ± 427	0.003^s^	1	0.01^s^
FFP	805 ± 309	998 ± 248	1070 ± 364	2695 ± 698	1	1	0.03^s^
**CLINICAL OUTCOMES**							
ICU LOS, days	3.8 ± 0.3	9.9 ± 0.8	14.5 ± 1.2	18.2 ± 1.8	<0.001^s^	<0.001^s^	0.09
Total LOS, days	9.8 ± 0.4	16.5 ± 1.0	21.8 ± 1.2	26.2 ± 2.1	<0.001^s^	0.001^s^	0.11
Mechanical ventilation, days	0.9 ± 0.2	5.5 ± 0.7	8.5 ± 1.0	13.3 ± 1.9	<0.001^s^	0.01^s^	0.002^s^
Nosocomial infection, *n* (%)	32 (16%)	41 (41%)	26 (49%)	19 (76%)	<0.001^s^	0.37	0.03^s^
Home-destination, *n* (%)	111 (56%)	34 (34%)	14 (26%)	5 (20%)	<0.001^s^	0.32	0.54

*Values are expressed as either n (%) or mean ± SEM*.

Fisher exact test or One-Way ANOVA used as appropriate with statistical significance set at

s *P < 0.05*.

### Disparate Contribution of Individual Organ Failure Components Among the Four Clusters

The four clusters differed not only in the average magnitude of MOD scores, but also in organ failure patterns (Supplemental Figure 1). Clusters 2–4 exhibited respiratory, cardiovascular, hematologic, and neurologic dysfunction scores that increased significantly between each severity-based cluster. Clusters 3 and 4 were distinguished from Clusters 1 and 2 by worse renal function. A notable increase respiratory and cardiovascular dysfunction scores was observed in Cluster 4 (Supplemental Figure 1). Therefore, organ dysfunction becomes progressively worse across the clusters for all systems except for the hepatic component, with a notable increase in average renal dysfunction scores in Cluster 3 and marked worsening of the severity of respiratory and cardiovascular dysfunction in Cluster 4.

### Distinct Inflammatory Patterns Emerge Among the Four Clusters

Seven out of the 31 biomarkers assayed exhibited statistically significant differences among the clusters upon admission and over time (Supplemental Figure 2). These included MCP-1, IL-6, IL-10, IL-8, IP-10, sST2, and MIG. The levels for these mediators at admission and over time are shown in Supplemental Figure 2 for each cluster. Cluster 2 was distinguished from Cluster 1 by significantly higher levels of MCP-1, IL-6, IL-10, IL-8, and sST2 early post-injury with notably sustained elevations in sST2 over 5 days. Clusters 3 and 4 exhibited further significant elevations in MCP-1, IL-6, IL-10, IL-8, and sST2 early and over time, as well as increases in IL-10 and MIG over clusters 1 and 2. The highest levels of all 7 mediators were seen in Cluster 4.

**Figure 3:**
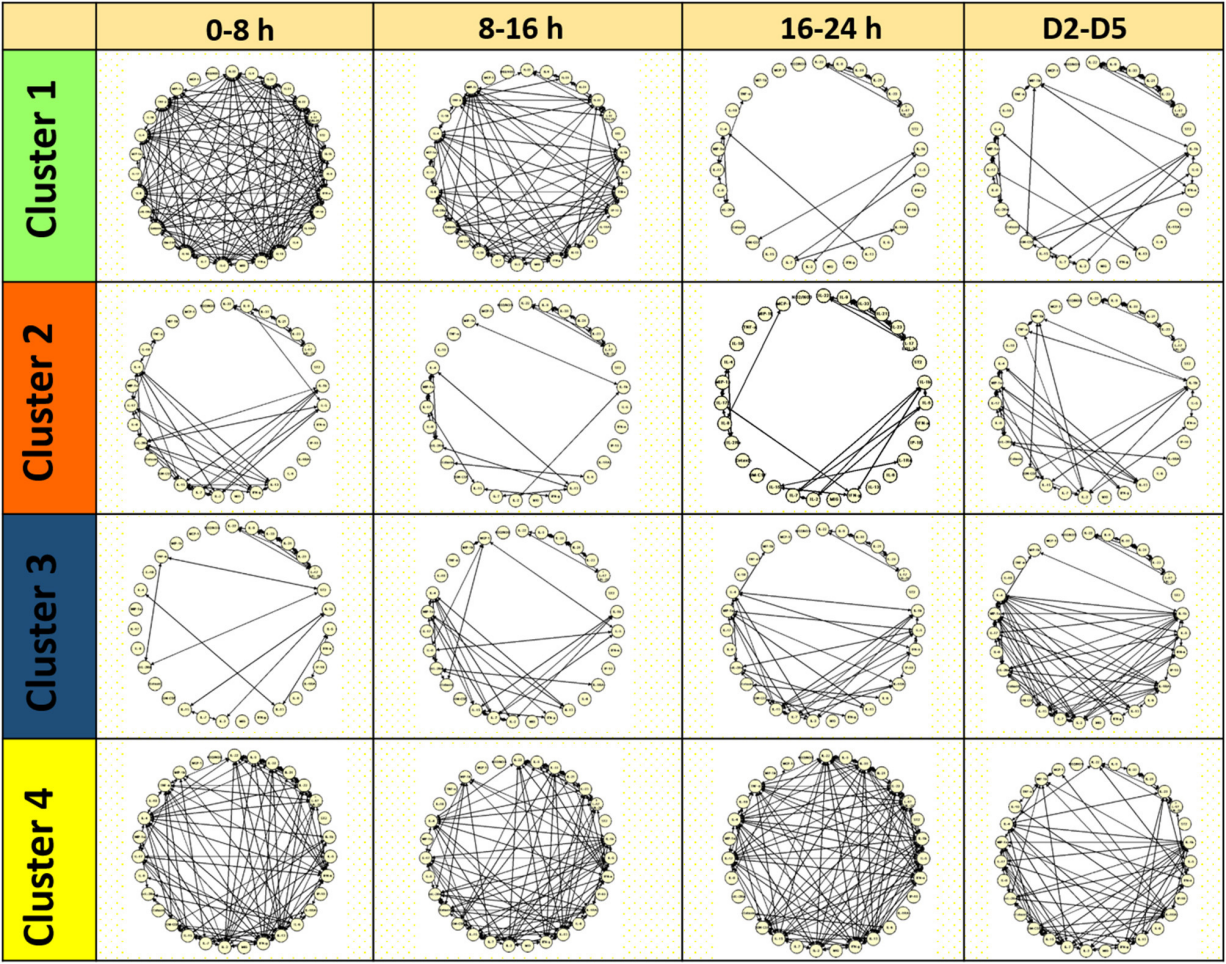
Dynamic Network analysis (DyNA) of inflammatory mediators among the four Clusters. DyNA suggests a differential inflammation profile in conjunction with MODS patterns.

Next, we sought to define the dynamic interconnectivity among different biomarkers across patients over different time intervals in the FCM-based MODS clusters. To do this, Dynamic Network Analysis (DyNA) was performed (see Materials and Methods). This analysis identifies interconnections among mediators across patients that exhibit dynamic changes in levels that correlate either positively or negatively. Figure 3 shows DyNA results for the four MODS clusters over four time intervals (0–8 h, 8–16 h, 16–24 h, and D2–D5). Notably, Cluster 1 exhibited a highly connected network within the first 16 h post-injury that dissipated rapidly thereafter. The networks in Cluster 2 consisted of sparsely connected networks, a pattern that persisted to day 5. In clear distinction, Cluster 3 had an increase in network connectivity over time with the greatest connectivity among mediators observed at D2–D5. Finally, Cluster 4 also exhibited a unique pattern with highly connected but uncoordinated networks throughout the 5 days. This analysis suggests that the inflammation profiles diverge early and in conjunction with the evolution of the severity and patterns of MODS following severe blunt trauma.

### Characteristics of Excluded Patient Cohorts

In order to assure availability of complete MOD score data from D2 to D5 and NI rates through discharge, patients with incomplete MOD D2–D5 data (*n* = 96) or that died in-hospital (*n* = 21) were not included in the initial clustering analysis. Among the 96 excluded patients, 91 of them were discharged from ICU prior to day 4, and their characteristics were comparable to patients in Cluster 1 (Supplemental Table 1). We have described previously the characteristics of the non-survivor cohort (21). To provide a comparison of the average MOD score values over time among the four clusters identified for survivors and the non-survivor cohort, we inserted the MOD score averages D2–D5 from the published non-survivor cohort with the curves for the survivor cohort (Supplemental Figure 3). It is interesting to note that MOD score values start on average lower in non-surviving patients than surviving patients in Clusters 3 and 4 but then rise steadily.

## Discussion

In the current study, we set out to identify organ dysfunction phenotypes in severely injured blunt trauma patients that survive to discharge using an unsupervised clustering strategy. The FCM followed by CVIs defined four distinct MODS patterns. These four clusters were not only different in the magnitude of MODS, but also in organ failure patterns exhibited by unique patterns of six Marshall MODS components. The clusters also differed both in the clinical features and inflammatory profiles upon admission and over time up to day 5 post-injury. This analysis suggests that it may be feasible to stratify critically ill trauma patients early in the clinical course into sub-groups at risk for multiple clinical trajectories defined by specific patterns and magnitude of organ dysfunction, which in turn may be useful in supporting tailored research and clinical therapies for blunt trauma patients.

Improvements in medicines have led to improved prognosis after severe injury (3, 26). Data from recent studies on large patient cohorts shows that MODS peaks within the first 5 days after injury (16, 20). Shepherd et al. (20) describe three broad patterns of organ function scores after injury including patients without MODS, and patients with either resolving MODS or non-resolving MODS. Others have defined a state of persistent critical illness characterized by prolonged ICU LOS associated with isolated organ dysfunction and risk for complications, including nosocomial infection (11, 13). These patients probably coincide with the non-resolving MODS in the work from Shepherd et al. (20).

In our study, we utilized a cohort of trauma patients deemed injured severely enough to be admitted to the ICU. We narrowed the cohort further to analyze data only from patients that remained in the ICU for at least 5 days to capture the 5 day “peak” for MODS and to assure that we included patients at risk for some degree of organ dysfunction. Others have shown adding MOD score information beyond day 5 may not add be useful to predicting MODS based phenotypes (27). However, this would require further analysis.

Using an unbiased clustering strategy, we determined that organ failure magnitude values between days 2 and 5 after injury fit into one of four distinct clusters. One of prime determinants of cluster destination is likely to be the injury and early shock characteristics. Cluster 1 was comprised of patients with moderate injury (average ISS = 18.7 ± 0.7) and minimal shock on presentation. ISS significantly increased in Cluster 2, indicating that Cluster 2 patients were more likely to have moderate-severe injury and, based on shock index, have some degree of shock; thus, these are defined as patients more likely to present with moderate-severe injury + mild shock/blood loss. Although ISS values were not different among Clusters 2–4, Cluster 3 patients had a higher likelihood of head and traumatic brain injury (TBI), and therefore were characterized by moderate-severe injury + mild shock + TBI. Cluster 4 included the patients who progressed to the highest MOD scores and had the same head injury rates as Cluster 3. These patients, despite having the same head injury rates as Cluster 3, had significantly higher transfusion requirements; therefore, this cluster appears to comprise patients presenting with moderate – severe injury + TBI + severe shock/blood loss. While some overlap in the presenting characteristics can be seen across the clusters, it is highly likely that the differences in presenting injury and early physiologic characteristics drive most of the subsequent organ dysfunction dynamics. Based in the prolonged ICU stays and complication rates observed in Clusters 3 and 4, it appears that these patient group would fall into the “non-resolving MODS” or “persistent critical illness” categories described by others (11, 13). Clusters 1 and 2 would likely fit into the phenotype of resolving MODS, with many of the patients in Cluster 1 exhibiting little evidence of MODS. However, our studies raise the possibility that even more precise designations than those described previously might be feasible based on MODS score magnitude and duration, or organ-specific responses.

Clinical manifestations of organ failure following trauma are diverse. Previous work examining the patterns of organ failure among individual trauma patients suggested a cumulative temporal sequence of single organ failures (28–30). Since the lungs are highly sensitive to mediator-induced inflammation, they are often the first system to show signs of failure (31–34). However, acute respiratory failure can precede MODS and may represent a trigger factor of subsequent dysregulated immune events that may contribute to remote organ failures (28, 29, 34). Although the typical sequence of organ failure in trauma patients is difficult to predict, the cardiovascular system is the second system to fail followed by renal failure (28, 35). It is apparent that there is a progressive increase in organ dysfunction with the increase in the MOD scores from cluster to cluster. While this is not surprising, our results show some interesting patterns, including a marked increase in hematologic and neurologic dysfunction between Clusters 1 and 2; a significant increase in the appearance of renal dysfunction in Clusters 3 and 4; and a large increase in the severity of cardiovascular and respiratory dysfunction in Cluster 4. Thus, there appears to be a hierarchy of organ dysfunction that may be driven in part by injury severity or pattern and the concomitant presence of shock and/or need for transfusion. Notably, age does not appear to a major distinguishing characteristic across the four clusters defined in the current study, despite multiple indications that age is a major complicating factor in trauma outcomes (6, 36, 37). This may be due in part, to the range of injury severity represented within our patient cohort. For example, we have recently shown that young and old patients that had moderate severity injury experienced a similar level of MODS (38). Two age-based sub-cohorts were identified, namely young (age: 18–30 years) and aged (age: 65–90 years) matched for ISS in the moderate range. Analysis of the average MOD score between these two groups showed that there were no statistically significantly differences with regards to the average MOD score between the two groups.

In other work, we have shown that levels of specific inflammatory mediators and DyNA patterns correlate with injury severity (39), hypotension (40), death (21), and complications such as nosocomial infection (8). Certain single nucleotide polymorphisms may also segregate patients at risk for organ dysfunction after trauma (41). Others have provided evidence that gene expression patterns in circulating leukocytes could be used to identify patients as risk for MOF after trauma (9, 42). Whether more precise stratification tools can be developed to prognosticate for the MODS clusters identified here is unknown. Our analysis of inflammation biomarker levels show clear differences the levels of certain inflammation biomarkers early and over time among the clusters. In addition, DyNA provides a visual window into the differences—and inferred degrees of coordination or lack thereof—in the dynamic changes in biomarker levels between the clusters over the early time periods following injury. Although significant differences in biomarker levels could be found among the clusters, we expect that levels of biomarkers alone may be insufficient to prognosticate for multiple clusters, and that other parameters as well as assessment of changes in biomarkers over time may be needed. For example, we have demonstrated the utility of patient-specific Principal Component Analysis based on inflammatory mediators assayed in the first 24 h following admission in differentiating later courses of MOD (43). In general, our observations point to stepwise increases in the magnitude and duration of the systemic inflammatory response with the MODS severity clusters.

As part of our inclusion criteria, we excluded patients died within first 24 h, and as expected the in-hospital mortality in the study cohort was low (21 out of 493 patients or 4.4%). We have previously reported the characteristics of the non-survivors (21), and found that the MODS trajectory was unique in these patients compared to those seen in the four clusters identified in the survivors. Although the average MOD score of the non-survivors would have segregated these patients into Cluster 2 or 3, the pattern was unique in that the MOD score started low and then steeply ascended. Whether patient destined to die during the initial hospitalization would constitute an additional MODS cluster will require additional work and larger patient numbers.

We have recently proposed a time window-based platform as a hypothetical construct for outcome stratification of trauma patients (44). This platform takes advantage of the fact that the time of the traumatic event is typically known, and facilitates adopting Precision Medicine methodology to quantify individualized injury response indices and thereby better prognosticate for adverse outcomes. We propose that optimization of this quantitative platform using MODS and subsets of MODS, i.e., cluster-based MODS, as a composite endpoint will lead to more informed early decision making, guide early interventions, improve quantifiable short- and long-term outcome indices, and could potentially facilitate tailored treatments or directed research.

There are several limitations to note in our study. First, this is a single institution study and 376 blunt trauma patients may not be sufficient to identify all of the organ failure phenotypes. Second, the study excluded blunt trauma non-survivors, and only patients with MOD score between D2 and D5 who survived to discharge were included. However, comparing the MOD score trajectory of the non-survivors to the four identified clusters in the survivor cohort suggests that non-survivors exhibit a distinct MODS pattern that can be differentiated from the four clusters by day 5. Third, our biomarker panel included only 31 inflammatory mediators that represent only a fraction of all potential circulating biomarkers. Finally, confirmatory studies involving multiple institutions that include contemporary patient data sets that incorporate penetrating trauma patients will be needed to confirm the number of MODS clusters.

## Data Availability Statement

The datasets generated for this study are available on request to the corresponding author.

## Ethics Statement

The studies involving human participants were reviewed and approved by University of Pittsburgh Institutional Review Board. The patients/participants or next of kin provided their written informed consent to participate in this study, as per IRB regulations.

## Author Contributions

DL: literature search, data analysis, and writing. RN: data collection, data analysis, and writing. YV: study design and critical revision. AP and RS: study design. HY: critical revision. QM: data analysis and data interpretation. TB: study design, data interpretation, and critical revision.

### Conflict of Interest

The authors declare that the research was conducted in the absence of any commercial or financial relationships that could be construed as a potential conflict of interest.

## Supplementary Material

Supplemental Figure 1 Patterns of specific organ dysfunction emerge among the four Clusters. Patients that fall in Clusters 2–4 exhibited greater dysfunction scores in the respiratory, cardiovascular, hematologic, and neurologic components when compared to Cluster 1 patients. Renal dysfunction was more prominent in Clusters 3 and 4 vs. Clusters 1 and 2. Cluster 4 patients exhibited a notable increase in respiratory and cardiovascular dysfunction scores when compared to the other three Clusters. ∽1 (cluster 1 vs. Cluster 2), ^§^(Cluster 2 vs. Cluster 3), ^*^(Cluster 3 vs. Cluster 4); *P* < 0.05 by One-Way ANOVA.

Supplemental Figure 2 Time course analysis of MCP-1, IL-6, IL-10, IL-8, IP-10, sST2, MIG, IL-22, IL-9, IL-33, IL-21, IL-23, IL-25, IL-1β, IL-5, IFN-α, IL-1RA, IL-13, IFN-γ, IL-2, IL-7, IL-15, GM-CSF, Eotaxin, sIL-2Rα, IL-17A, MIP-1α, IL-4, TNF-α, MIP-1β, and ${\text{NO}}_{2}^{-}$/${\text{NO}}_{3}^{-}$ among the four clusters from time of injury up to day 5 compared to the healthy volunteers (*n* = 12). Data presented as mean ± SEM. ∽1 (cluster 1 vs. Cluster 2), ^§^(Cluster 2 vs. Cluster 3), ^*^(Cluster 3 vs. Cluster 4); *P* < 0.05 by Two-Way ANOVA.

Supplemental Figure 3 Average MOD scores of the four FCM-derived Clusters compared to the average MOD score of the non-survivor patients (*n* = 21) from day 2 through day 5 post-injury. Data presented as mean with 95% confidence interval (CI).

Supplemental Table 1 Comparison of demographic and injury pattern characteristics, admission physiological and biochemical parameters between Cluster 1 patients (*n* = 199) and the excluded survivor patients (*n* = 96). Values are expressed as mean ± SEM. Mann-Whitney *U*-test, One-Way ANOVA, and Fisher exact test were used as appropriate with statistical significance set at *P* < 0.05.

## Abbreviations

MODS: multiple organ dysfunction syndrome
ISS: injury severity score
ICU: intensive care unit
IL: interleukin
DyNA: dynamic network analysis.
