# Supplementary figures and images for: Unsupervised Clustering Analysis Based on MODS Severity Identifies Four Distinct Organ Dysfunction Patterns in Severely Injured Blunt Trauma Patients
Source: Front Med (Lausanne). 2020 Feb 25;7:46. doi: 10.3389/fmed.2020.00046 (PMC7053419; doi:10.3389/fmed.2020.00046)

**Supplemental Fig.1**


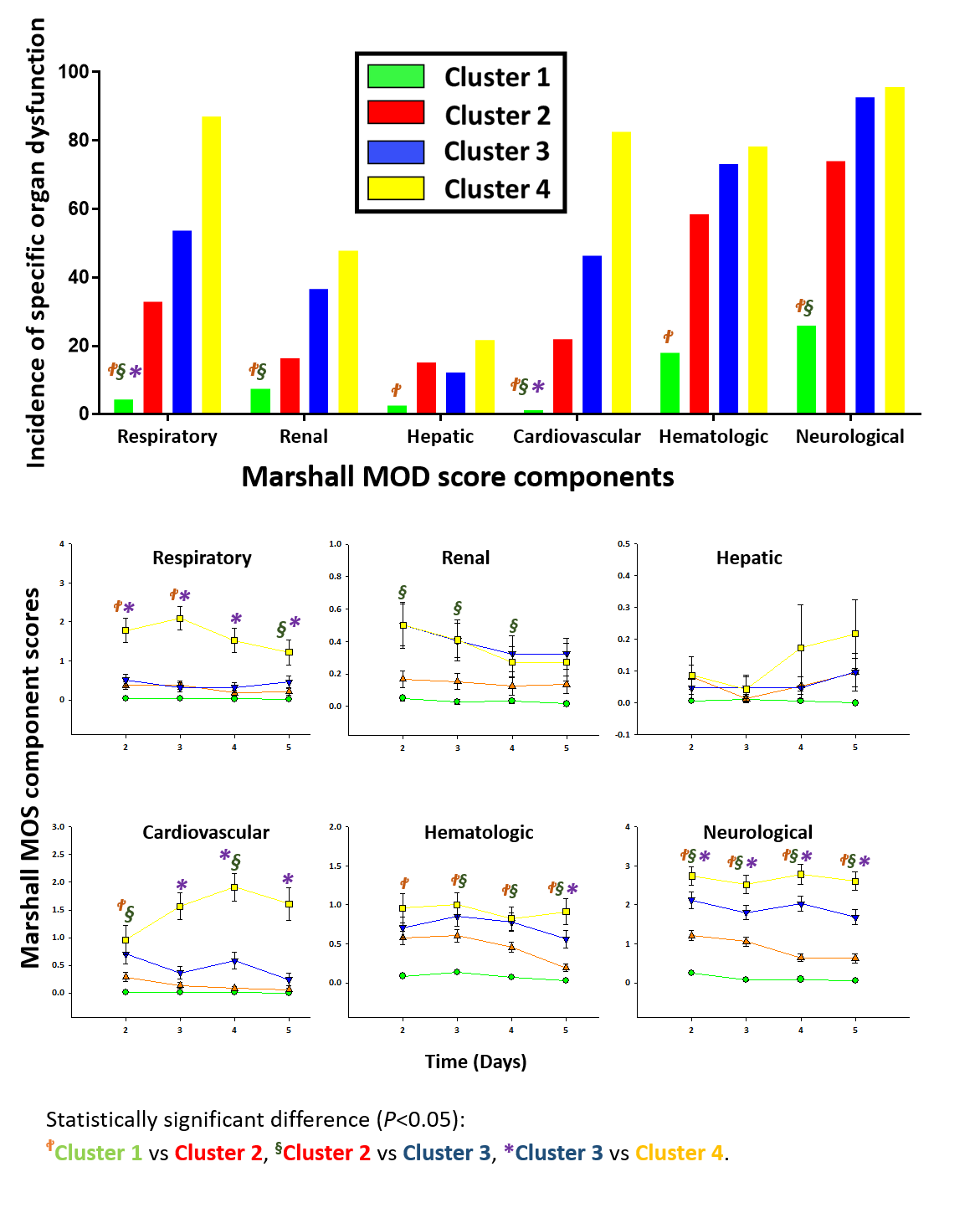


**
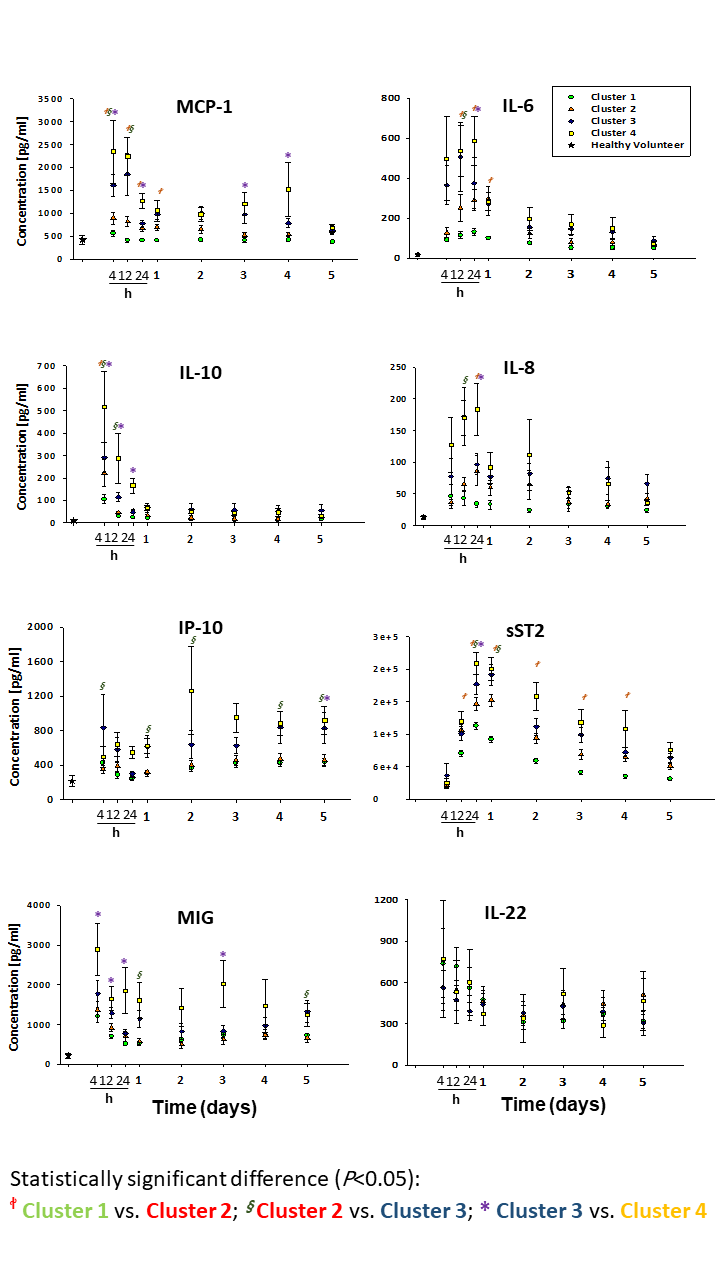
Supplemental Fig.2**

**
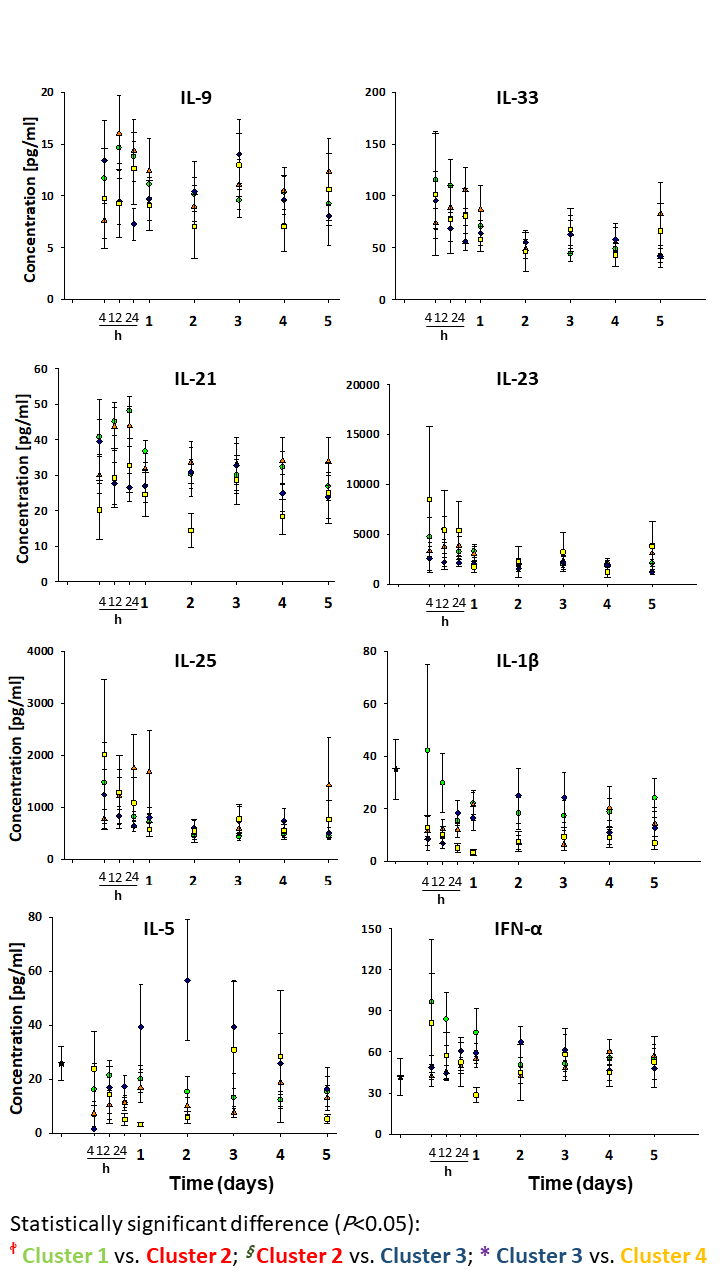
**

**
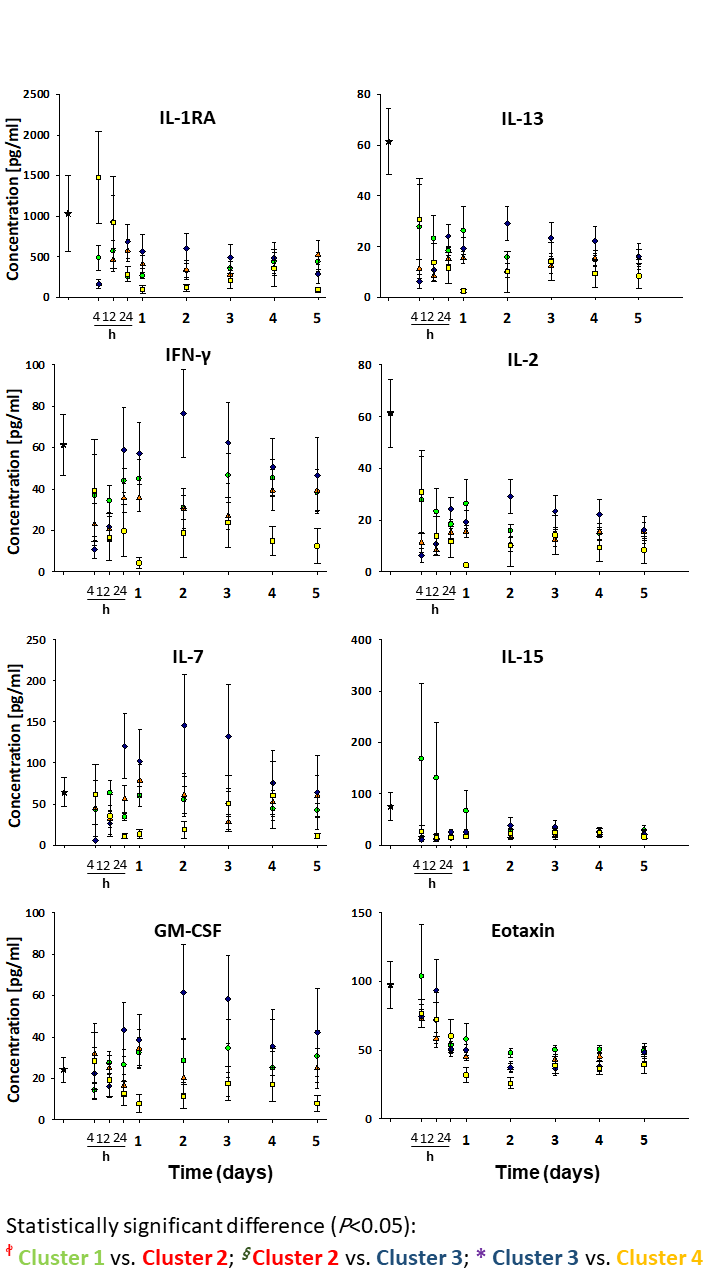
**

**
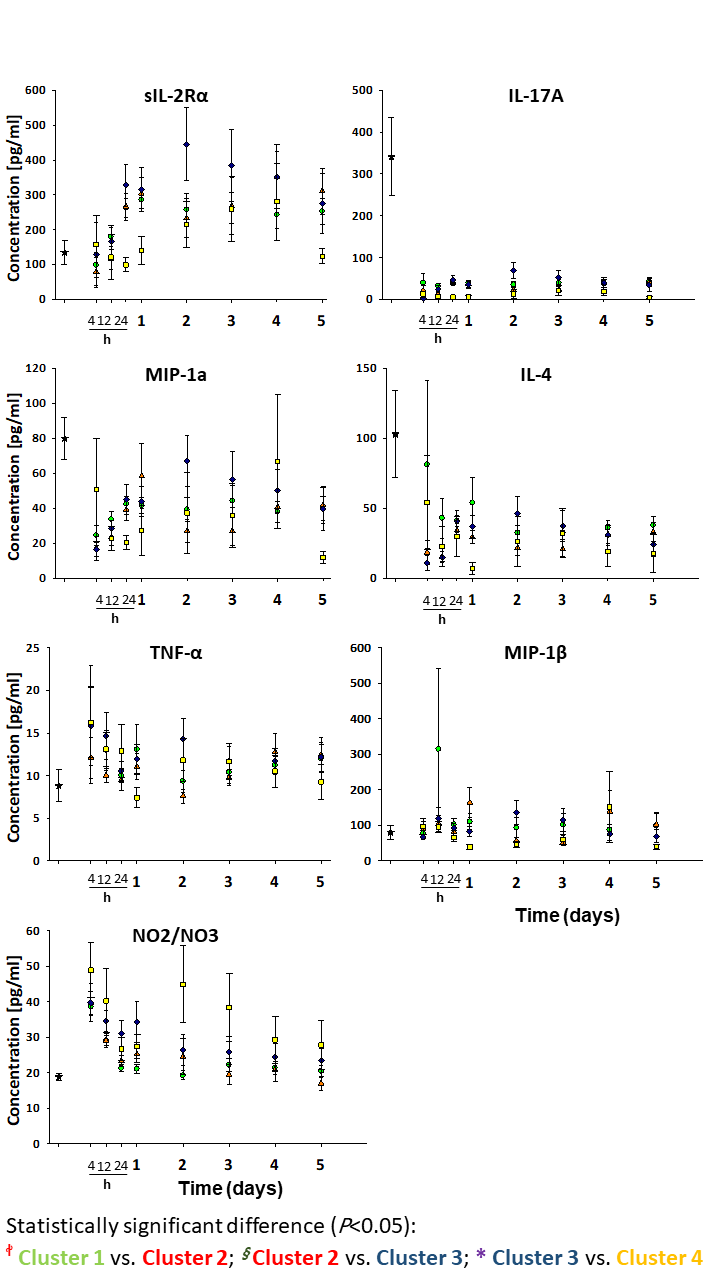
Supplemental Fig.3**

**
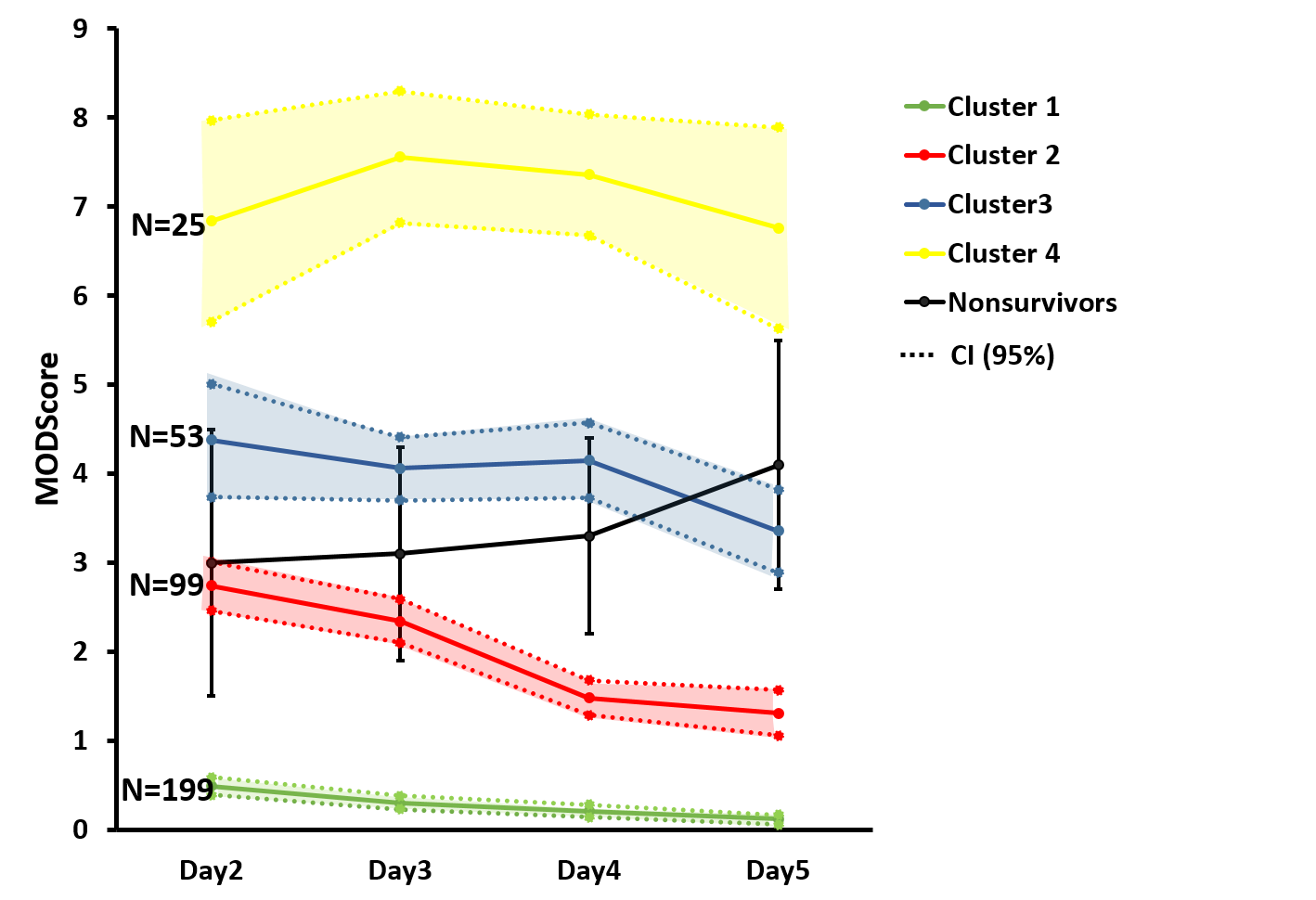
**

Supplement: Supplemental Figure 1 — Patterns of specific organ dysfunction emerge among the four Clusters. Patients that fall in Clusters 2–4 exhibited greater dysfunction scores in the respiratory, cardiovascular, hematologic, and neurologic components when compared to Cluster 1 patients. Renal dysfunction was more prominent in Clusters 3 and 4 vs. Clusters 1 and 2. Cluster 4 patients exhibited a notable increase in respiratory and cardiovascular dysfunction scores when compared to the other three Clusters. ∽1 (cluster 1 vs. Cluster 2), §(Cluster 2 vs. Cluster 3), *(Cluster 3 vs. Cluster 4); P < 0.05 by One-Way ANOVA. [file Data_Sheet_1.docx]
