# Supplementary material for: Unsupervised Clustering Analysis Based on MODS Severity Identifies Four Distinct Organ Dysfunction Patterns in Severely Injured Blunt Trauma Patients
Source: Front Med (Lausanne). 2020 Feb 25;7:46. doi: 10.3389/fmed.2020.00046 (PMC7053419; doi:10.3389/fmed.2020.00046)
Supplement: Supplemental Table 1 — Comparison of demographic and injury pattern characteristics, admission physiological and biochemical parameters between Cluster 1 patients (n = 199) and the excluded survivor patients (n = 96). Values are expressed as mean ± SEM. Mann-Whitney U-test, One-Way ANOVA, and Fisher exact test were used as appropriate with statistical significance set at P < 0.05. [file Table_1.docx]

**Supplemental Table 1**

|  | **Cluster 1 (n=199)** | **Excluded survivors**  **(n=96)** | ***P* value** |
| --- | --- | --- | --- |
|  |  |  |  |
| ***Demographics*** | | | |
| Age, yr | 49.4 ± 1.3 | 48.3 ± 2.1 | 0.66 |
| Gender, M/F | 134/65 | 70/26 | 0.35 |
| Injury Severity Score (ISS) | 18.7 ± 0.7 | 14.1 ± 0.8 | <0.001 |
| ***Abbreviated Injury Scale (AIS)*** | | | |
| S1 (head/neck) | 1.19 ± 0.12 | 1.45 ± 0.18 | 0.22 |
| S2 (face) | 0.42 ± 0.06 | 0.34 ± 0.08 | 0.44 |
| S3 (chest) | 2.17 ± 0.11 | 1.22 ± 0.15 | <0.001 |
| S4 (abdomen) | 1.02 ± 0.10 | 0.73 ± 0.12 | 0.08 |
| S5 (extremities) | 1.36 ± 0.09 | 1.04 ± 0.15 | 0.06 |
| S6 (external) | 0.69 ± 0.04 | 0.63 ± 0.05 | 0.38 |
| ***Physiological and Biochemical parameters within 24 h*** | | | |
| Heart rate | 93.1 ± 1.5 | 95.4 ± 2.3 | 0.47 |
| Systolic blood pressure | 130.1 ± 1.9 | 140.5 ± 2.7 | 0.002 |
| Shock Index | 0.8 ± 0.1 | 0.7 ± 0.0 | 0.17 |
| Base deficit (BD) | 4.6 ± 0.4 | 4.1 ± 0.6 | 0.45 |
| Lactate | 2.7 ± 0.2 | 2.9 ± 0.4 | 0.49 |
| Creatinine phosphokinase (CPK) | 1047 ± 177 | 1089 ± 308 | 0.9 |
| Sodium (Na) | 138.2 ± 0.2 | 138.3 ± 0.3 | 0.6 |
| Potassium (K) | 4.0 ± 0.0 | 3.9 ± 0.1 | 0.21 |
| Chloride (Cl) | 105.6 ± 0.3 | 104.8 ± 0.4 | 0.17 |
| Partial pressure of arterial carbon dioxide (PaCO2) | 23.4 ± 0.2 | 24.1 ± 0.4 | 0.1 |
| Anion gap | 10.2 ± 0.3 | 11.0 ± 1.1 | 0.32 |
| Blood urea nitrogen (BUN) | 14.5 ± 0.4 | 13.8 ± 1.1 | 0.46 |
| Creatinine | 1.01 ± 0.05 | 1.01 ± 0.06 | 0.97 |
| Glucose | 152.7 ± 4.2 | 137.8 ± 4.8 | 0.03 |
| Albumin | 3.5 ± 0.1 | 3.4 ± 0.1 | 0.45 |
| Total protein | 5.9 ± 0.2 | 5.8 ± 0.2 | 0.81 |
| Bilirubin total | 0.8 ± 0.1 | 0.9 ± 0.1 | 0.59 |
| Bilirubin direct | 1.4 ± 1.2 | 0.2 ± 0.1 | 0.52 |
| Alanine amino-transferase (ALT) | 111.4 ± 21.0 | 103.1 ± 43.0 | 0.85 |
| Aspartate amino-transferase (AST) | 148.5 ± 29.4 | 147.6 ± 73.1 | 0.99 |
| White blood cell count | 15.4 ± 0.4 | 14.1 ± 0.7 | 0.13 |
| Hemoglobin | 13.3 ± 0.1 | 13.4 ± 0.2 | 0.59 |
| Hematocrit | 38.7 ± 0.4 | 39.1 ± 0.5 | 0.53 |
| Platelets | 244.8 ± 5.1 | 220.9 ± 7.0 | 0.007 |
| Neutrophils | 75.7 ± 0.8 | 75.7 ± 1.5 | 0.98 |
| Lymphs | 14.9 ± 0.7 | 15.1 ± 1.2 | 0.9 |
| Monocytes | 5.8 ± 0.2 | 6.1 ± 0.3 | 0.31 |
| Eosinophils | 0.96 ± 0.08 | 1.00 ± 0.15 | 0.79 |
| Basophils | 0.17 ± 0.03 | 0.26 ± 0.05 | 0.1 |
| Prothrombin time (PT), seconds | 14.4 ± 0.2 | 14.3 ± 0.3 | 0.76 |
| International normalized ratio (INR) | 1.16 ± 0.02 | 1.17 ± 0.03 | 0.94 |
| Partial thromboplastin time (PTT), seconds | 26.3 ± 0.3 | 26.6 ± 0.4 | 0.54 |
